# Development of an instrument-free and low-cost ELISA dot-blot test to detect antibodies against SARS-CoV-2

**DOI:** 10.1515/biol-2022-0577

**Published:** 2023-08-09

**Authors:** Navilla Apú, Germán Madrigal-Redondo, María Herrera Vega, Eugenia Corrales-Aguilar, Ismael Segura-Ulate

**Affiliations:** Facultad de Farmacia, Instituto de Investigaciones Farmacéuticas (INIFAR), Universidad de Costa Rica, San José, Costa Rica; Facultad de Microbiología, Instituto Clodomiro Picado (ICP), Universidad de Costa Rica, San José, Costa Rica; Facultad de Microbiología, Centro de Investigación en Enfermedades Tropicales (CIET), Universidad de Costa Rica, San José, Costa Rica

**Keywords:** SARS-CoV-2, COVID-19, immunity, antibodies, seroprevalence, ELISA dot-blot

## Abstract

Most laboratory tests to detect the presence of anti-SARS-CoV-2 antibodies use enzyme-linked immunosorbent assays (ELISA) or chemiluminescence immunoassays (CLIA); however, equipment for these immunoassays is unavailable in many areas of low- and middle-income countries. Rapid lateral flow immunoassay (LFIA) tests are an equipment-free option, but their high price may make them less suitable for conducting seroprevalence surveys. Here, we describe a simple dual antigen ELISA dot-blot test to detect anti-SARS-CoV-2 IgG antibodies with high sensitivity (94–98%) and specificity (92–100%), compared to commercially available ELISA and CLIA options. Additionally, this ELISA dot-blot test can be completed in one hour using minimal laboratory equipment. Importantly, this immunoassay is significantly more affordable than most LFIA tests available on the global market. The dot-blot strips may be stored for up to 7 days under freezing conditions. This ELISA dot-blot test is a cost-effective option for conducting seroprevalence screenings in areas lacking ELISA or CLIA facilities, compared to LFIA tests.

## 1 Introduction

While official corona virus disease 2019 (COVID-19) cases have accumulated worldwide, it is possible that an even larger number of undetected COVID-19 cases have escaped the official toll [1,2]. Furthermore, in countries where healthcare systems lack the resources to effectively handle the pandemic, including for epidemiological surveillance, the ratio of undetected to official cases is likely to be even higher. This is particularly true in many areas of low- and middle-income countries, where inadequate testing has been the norm since the beginning of the pandemic [3–5].

Although the mechanisms and consequences of anti-SARS-CoV-2 immunity resulting from previous infection or variants are not yet fully understood, pre-omicron evidence suggests that reinfection cases occur at a much lower rate than first infections, and reinfections are associated with a milder course [6–8]. Thus, as more and more people have overcome COVID-19, seroprevalence data become a key component in epidemiological and policy decision-making. For example, having a clear understanding of seroprevalence data broken down by groups and areas allows for identification of populations with the largest numbers of immunologically naive individuals, which can inform decision-making, and tailor decisions based on the population’s potential risks for new outbreaks, waves, or viral variants. It has been suggested that in areas with high seroprevalence and limited resources, the focus should shift from preventing infections to addressing other urgent needs related to the pandemic [9]. Unfortunately, the same limitations that led to low testing rates during the pandemic may also hinder seroprevalence surveillance efforts, particularly in areas without access to facilities equipped to perform enzyme-linked immunosorbent assays (ELISA) or chemiluminescence immunoassays (CLIA), the two most common types of immunoassays to detect seroconversion. Rapid lateral flow immunoassays (LFIA) offer an equipment-free option, but their high cost can be prohibitive for healthcare systems with limited resources.

In this study, we present an ELISA dot-blot test to detect anti-SARS-CoV-2 antibodies in human samples. This immunoassay format only requires basic laboratory equipment, including micropipettes, centrifuges, and shakers. Additionally, this test is cost-effective, requiring consumables that are a fraction of the cost of the most affordable LFIA tests available on the global market. The turnaround time for this ELISA dot-blot test is reasonable, taking approximately one hour to complete, with an additional hour required for preparing the dot-blot strips. Finally, this ELISA dot-blot test also has high sensitivity and specificity when compared to commercial ELISA or CLIA as comparison standards.

## 2 Materials and methods

### 2.1 Immunoblot evaluation of recombinant proteins

Fifty nanograms of recombinant SARS-CoV-2 receptor binding domain (RBD) or nucleocapsid proteins (Cat. # 40592-V08H7 and Cat. # 40588-V08B, Sino Biologicals; Beijing, China) dissolved in sterile phosphate buffer saline (PBS) were mixed with 2× sodium dodecyl sulfate (SDS) loading buffer and boiled for 10 min. Each recombinant protein was loaded in 12% polyacrylamide gels, separated by SDS-polyacrylamide gel electrophoresis (PAGE), and transferred to a nitrocellulose membrane. Membranes were blocked in 5% non-fat milk dissolved in PBS with 0.1% Tween 100 (PBS-Tween) and incubated overnight at 4 °C with anti-His-tag primary antibody (Cat. # MA1-21315, Thermo Fisher Scientific; Waltham, MA, USA) at a 1:1,000 dilution or a plasma pool of positive COVID-19 patients at a 1:500 dilution. Secondary antibodies included HRP-conjugated goat anti-human IgG (anti-IgG) or IgM (anti-IgM) secondary antibodies (Cat. # 2040-05 and 2020-01, Southern Biotech; Birmingham, AL, USA) at a 1:5,000 dilution. Immunoblotting was revealed using Western Blotting Luminol Reagent (Cat. # Sc-2048, Santa Cruz Biotechnology; Dallas, TX, USA) and visualized with a ChemiDoc Gel Imaging System (Bio-Rad; Hercules, CA, USA).

### 2.2 Preparation of dot-blot membrane strips

Lyophilized recombinant SARS-CoV-2 RBD and nucleocapsid proteins were dissolved in sterile PBS to a final concentration of 250 ng/µL following the manufacturer’s instructions. Bovine albumin was also prepared in sterile PBS at a final concentration of 250 ng/µL.

Small nitrocellulose membrane strips were cut into a rectangular shape measuring approximately 0.7 × 2.1 mm. We recommend cutting longer strips if a labeling area is required. The strips were divided into three separate squares, each measuring approximately 0.7 × 0.7 mm, using a thin permanent marker. Protein spots for albumin, RBD, and nucleocapsid proteins were, respectively, fixed onto the center of each of the three squares.

Proteins were spotted onto the nitrocellulose membrane strips using a 0.5–10 µL micropipette. Well-defined protein spots were created by pipetting 0.8 µL of protein solution, which is equivalent to 200 ng of protein. The spotting procedure was performed twice at each position, resulting in a total of 400 ng of fixed protein at each antigen location. After dispensing the first protein load (200 ng) onto the membrane, we waited for at least 30 s to allow the PBS to diffuse across the membrane, before dispensing the second protein load (subsequent 200 ng).

Proper protein fixation and spot distribution were visualized on the membrane strips by quickly immersing them in Ponceau stain for 1 min, followed by two 1-minute washes with ample distilled water. This Ponceau staining is an optional step that can be used as a quality control measure to confirm proper protein loading and antigen spot distribution (Figure 2).

Directly following Ponceau staining, the strips were blocked on a shaker with a 5% non-fat milk solution in PBS-Tween for 30 min at room temperature (RT). The membrane-blocking step was followed by a single wash with ample PBS-Tween for 5 min on a shaker. The membrane strips were then dried quickly on a clean paper towel, with the top facing up, and used for testing within 60 min of preparation.

### 2.3 Human COVID-19 convalescent plasma and testing with commercial CLIA and ELISA standards

Human convalescent plasma was voluntarily donated by 59 RT-qPCR confirmed COVID-19 recovered patients who provided informed consent to have their samples tested for the presence of anti-SARS-CoV-2 antibodies. Plasma from each of the 59 confirmed COVID-19 cases was tested with MAGLUMI 2019-nCoV (SARSCoV-2) IgG kit (Cat. # SNB-130219016M, Snibe Diagnostics; Shenzhen, China) that detects immunoreactivity against both SARS-CoV-2 full-length Spike protein (which includes the RBD protein) and nucleocapsid protein. Likewise, plasma samples from 43 of these COVID-19-confirmed cases were also tested with the anti-SARS-CoV-2 ELISA IgG kit (Cat. # EI 2606-9601 G, EUROIMMUN; Lübeck, Germany), which detects immunoreactivity against SARS-CoV-2 RBD protein alone. All CLIA and ELISA tests were performed according to the manufacturer’s instructions.

Pre-pandemic plasma samples from 20 individuals were also tested. These individuals provided an informed consent to have their plasma tested for immunoreactivity against different viruses, including dengue, zika, chikungunya, and emerging diseases.


**Informed consent:** Informed consent has been obtained from all individuals included in this study.
**Ethical approval:** The research related to human use has been complied with all the relevant national regulations, and institutional policies and in accordance with the tenets of the Helsinki Declaration, and has been approved by the authors’ institutional review board or equivalent committee.

### 2.4 Dot-blot membrane strips testing with human plasma and monoclonal antibody

Membrane strips spotted with the antigens (RBD and nucleocapsid proteins) and negative control (albumin) proteins were incubated on a shaker with COVID-19 convalescent plasma, as well as pre-pandemic plasma samples for 30 min at RT. All plasma samples were diluted to 1:500 in 5% non-fat milk in PBS-Tween. It is possible to test multiple plasma samples individually by using sealable plastic sleeves for each strip during the incubation; however, we found that the most convenient method is to place each strip along with its corresponding plasma solution inside a 2-mL Eppendorf tube. A single dot-blot strip was incubated with an anti-RBD protein monoclonal antibody (NR-52481 clone CR3022 produced in HEK293 cells under HHSN272201400008C and obtained through BEI Resources, NIAID, NIH: Monoclonal Anti-SARS Coronavirus Recombinant Human Antibody). This anti-RBD antibody was used at a 1:1,000 dilution in 5% non-fat milk in PBS-Tween. After incubation with the plasma or monoclonal antibody solutions, strips were washed in a single step with abundant PBS-Tween for 5 min. Membrane strips were then incubated on a shaker with anti-IgG secondary antibody diluted to 1:5,000 in 5% non-fat milk in PBS-Tween for 30 min at RT.

After incubation, dot-blot strips were washed in a single step with abundant PBS-Tween for 5 min. The membrane strips were then quickly dried, with the top facing up, on a clean paper towel and place on a clean hydrophobic surface such as Parafilm M. Each strip was covered with approximately 200 µL of 3,3′,5,5′-tetramethylbenzidine (TMB) Liquid Substrate System for Membranes (Cat. # T0565, Sigma-Aldrich; St. Louis, MO, USA) for 2 min and excess TMB was removed with a quick submersion in distilled water. After chromogenic development, the membrane strips were quickly dried, with the top facing up, on a clean paper towel and the results were either assessed by eye and documented within the next hour or photographed with a cell phone camera to evaluate the results afterwards.

### 2.5 Dot-blot strips stability under storage conditions

Freshly prepared ELISA dot-blot membrane strips, as described in the “Preparation of dot-blot membrane strips” section, were sealed inside plastic sleeves with enough PBS-Tween to keep them damp. These sealed strips were then stored for 3 or 7 days at 4 or −10°C. After these periods, the strips were tested with COVID-19-positive plasma pool as described in the “Dot-blot membrane strips testing with human plasma and monoclonal antibody” section. A freshly prepared dot-blot strip was also included for each of these tests as a comparison standard for the functionality of the stored strips.

### 2.6 Statistical analysis

Sensitivity, specificity, and predictive diagnostic values of the ELISA dot-blot test were determined using a Fisher’s exact test specifically designed for this type of evaluation. This statistical test is included in the GraphPad Prism 8 software. For this statistical analysis, the performance parameters of the test in evaluation were compared against the results of a commercially available ELISA or CLIA. Both commercially available assays were used as the comparison standard required for this type of analysis. All other analysis options were kept as default.

## 3 Results

**Figure 1:**
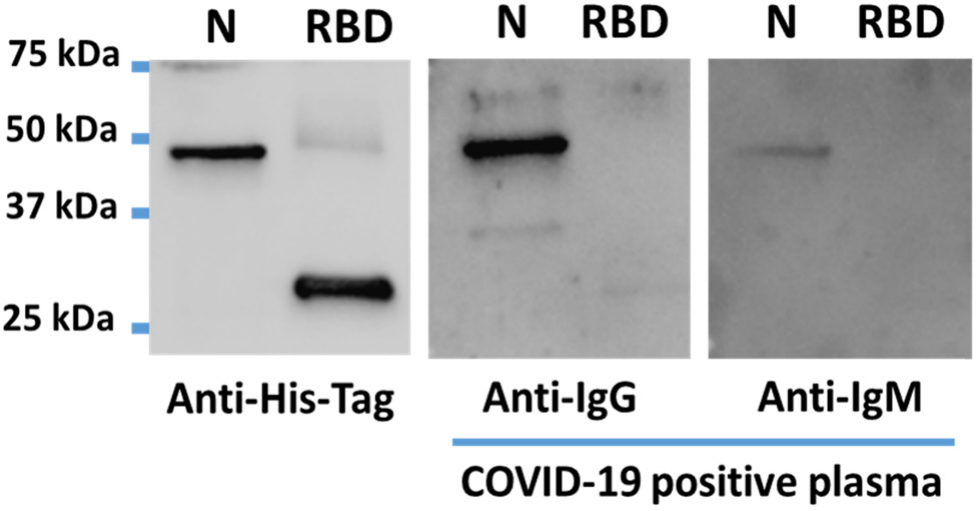
Representative immunoblotting images of recombinant SARS-CoV-2 nucleocapsid (N) and RBD proteins. From left to right: immunodetection with an anti-His-tag antibody shows clear bands for both nucleocapsid and RBD recombinant proteins at their expected molecular sizes; human COVID-19-positive plasma pool plus an anti-IgG antibody detects a clear nucleocapsid protein band, but no RBD is observed; human COVID-19-positive plasma pool plus an anti-IgM antibody detects a faint nucleocapsid protein band and no RBD band at all.

Immunoblotting of the His-tagged recombinant viral proteins using an anti-His-tag antibody revealed clear bands for each protein at approximately their predicted molecular weights of 47 kDa for nucleocapsid protein and 27 kDa for RBD protein. However, when tested with a COVID-19-positive plasma pool, only the nucleocapsid protein band was detected (Figure 1). Immunoblotting with the COVID-19-positive plasma pool also revealed differences between IgG and IgM immunoreactivity. In this case, there was a clear nucleocapsid protein band when using anti-IgG detection, but only a barely visible band when using anti-IgM secondary antibodies (Figure 1).

**Figure 2:**
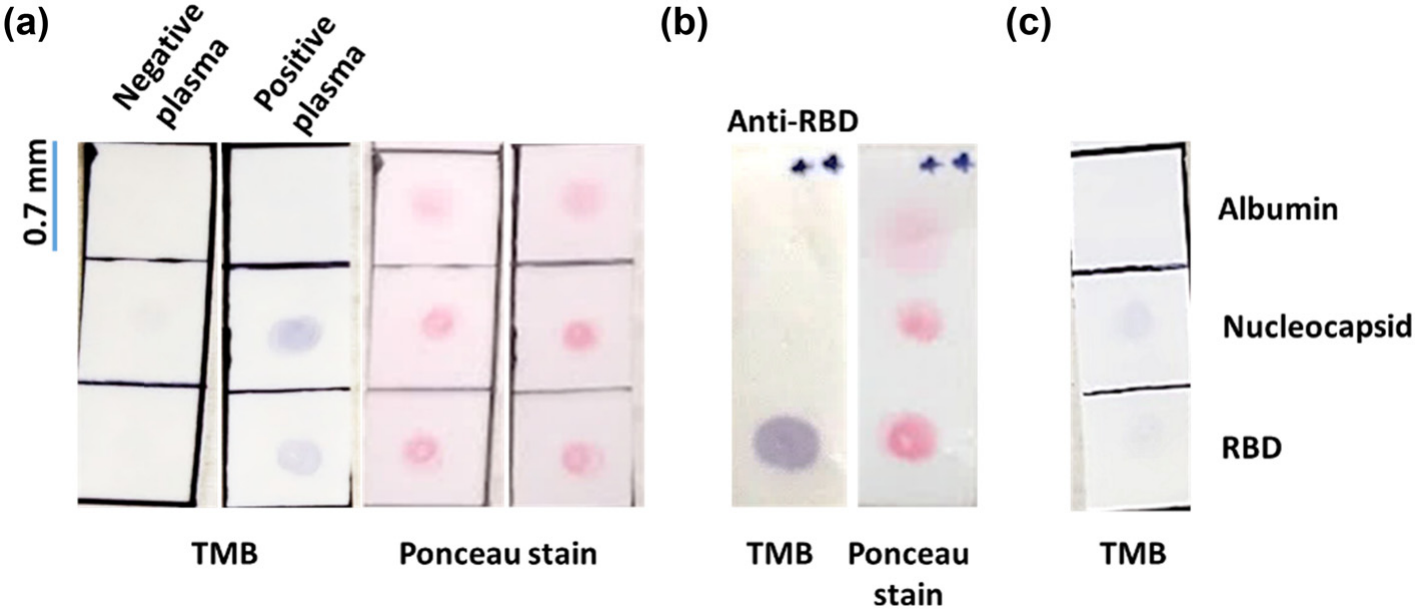
Representative photographic images of Ponceau-stained dot-blot strips showing protein loading and distribution (right side), as well as ELISA dot-blot strips from finalized tests developed with blotting TMB (left side). (a) Dot-blot strip incubated with COVID-19-positive plasma plus an anti-IgG secondary antibody reveals purple dots at both the nucleocapsid and RBD protein positions. No chromogenic signal is noticeable at any of the antigen positions of the strip incubated with COVID-19-negative plasma plus an anti-IgG secondary antibody. (b) Monoclonal anti-RBD antibody produces a clear immunodetection at the RBD protein spot, with no chromogenic signal at the nucleocapsid protein or albumin positions. (c) Illustrative dot-blot strip showing a single weak chromogenic spot at the nucleocapsid protein position and no purple color at the RBD protein or albumin positions.

The interpretation of the ELISA dot-blot strip results was conducted as follows: any membrane strip that had a visible and well-defined purple dot at one or both of the SARS-CoV-2 antigen positions (Figure 2) was considered positive for anti-SARS-CoV-2 antibodies, unless the albumin-negative control spot had turned purple as well. A single weak purple spot at either the RBD or nucleocapsid proteins positions was considered negative for anti-SARS-CoV-2 antibodies (e.g., the nucleocapsid protein position shown in Figure 2c). Although no samples with a double weak purple spot were observed at the RBD and nucleocapsid proteins positions, such a finding would have been considered an indeterminate result that requires to be retested. In addition, in any cases for which the albumin-negative control spot turned purple, the test was repeated. However, only 3 of 59 plasma samples from COVID-19 cases (roughly 4%) required repeated testing, and none of the 20 pre-pandemic samples required such repetition. Samples without any purple spots were considered negative for anti-SARS-CoV-2 antibodies.

**Figure 3:**
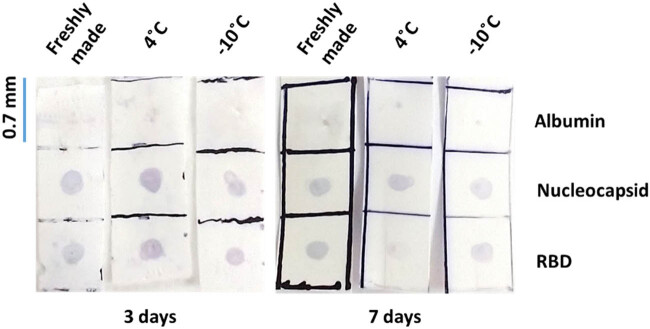
Representative photographic images of dot-blot strips stored under different conditions, incubated with COVID-19-positive plasma plus an anti-IgG secondary antibody, and revealed using blotting TMB. Results of a freshly prepared dot-blot strip compared to strips stored in PBS-Tween for 3 days at 4 or −10°C. Images show that the stored strips fully conserved immunoreactivity to detect anti-SARS-CoV-2 antibodies in human plasma. Also, the results of a freshly prepared dot-blot strip compared to strips stored in PBS-Tween for 7 days at 4 or −10°C. Images show a substantial loss of immunoreactivity against RBD protein after storage at 4°C; nonetheless, immunoreactivity against both antigens was preserved after storage at −10°C.

The stability tests of the ELISA dot-blot membrane strips stored at 4°C or −10°C for 3 days demonstrated that their immunoreactivity properties remained fully functional under these storage conditions (Figure 3). When we extended the time to 7 days under the same storage conditions, we found that the strips stored at 4°C lost their anti-RBD immunoreactivity properties; nonetheless, strips stored at −10°C remained fully functional.

Detection of RBD protein in the ELISA dot-blot strip using a monoclonal anti-RBD antibody produced a strong signal in the RBD protein spot with no unspecific signal detected at the nucleocapsid protein or albumin spots (Figure 2b). The incubation of the ELISA dot-blot strips with COVID-19-positive plasma pool and anti-IgG antibodies produced well-defined purple spots both at the nucleocapsid and at RBD proteins positions, with a slightly stronger signal at the nucleocapsid protein spot (Figure 2a). Some COVID-19-positive samples produced a single purple spot at the nucleocapsid protein position, without any chromogenic signal at the RBD protein position (not shown). However, COVID-19-negative plasma pool did not produce any purple spots at RBD protein, nucleocapsid protein, or albumin positions (Figure 2a). Importantly, we observed no purple spots at RBD protein, nucleocapsid protein, or albumin positions from any of the 20 pre-pandemic plasma samples.

Fisher’s exact test comparing the ELISA dot-blot with the commercial ELISA standard resulted in a 94% sensitivity and 100% specificity (Table 1). The positive predictive value and negative predictive values for this comparison were 100 and 94%, respectively. Similarly, comparison against the commercial CLIA standard resulted in a 98% sensitivity and 92% specificity (Table 1). In this latter case, the positive predictive value and negative predictive value were 93 and 97%, respectively.

**Table 1:** Fisher’s exact test results for sensitivity and specificity comparing the ELISA dot-blot test against two different commercially available immunoassays used as comparison standards: an ELISA (Snibe Diagnostics) and a CLIA (EUROIMMUN)

	ELISA			CLIA		
	Sample size	Positive samples	Negative samples	Sample size	Positive samples	Negative samples
ELISA dot-blot positive samples	*N* = 29	29	0	*N* = 38	35	3
ELISA dot-blot negative samples	*N* = 33	2	31	*N* = 41	1	40
Specificity	100%			92%		
Sensitivity	94%			98%		
*p*-Value	<0.0001			<0.0001		

## 4 Discussion

From its inception, our effort has been focused on developing an easy-to-implement, cost-effective immunoassay for the detection of anti-SARS-CoV-2 antibodies that can be used by healthcare systems at any location, even those lacking access to a clinical laboratory. We considered that in order to accomplish its main objective, our assay should satisfy four key requirements: i) its protocol must be free of costly instruments, ii) all necessary consumables must be commercially available, iii) its preparation and testing protocols must be simple, and iv) the overall cost of the consumables ought to be considerably less than the price of commercially available LFIA tests. In our opinion, the ELISA dot-blot was the only technique that could meet all these criteria. Similar approaches have been devised in the past; for example, Chow et al. [10] detected SARS seroconversion among healthcare workers with nearly perfect specificity and sensitivity using an ELISA dot-blot assay. However, their test used heat-inactivated SARS virions as antigens, which required a highly specialized BSL-3 laboratory to produce the main component. In contrast, we decided to utilize commercially available recombinant SARS-CoV-2 proteins as antigens, as they can be easily obtained from various vendors worldwide.

In accordance with our established criteria, we developed an alternative protein spotting protocol to avoid the use of a dot-blot protein loading apparatus. This protocol enabled the production of clearly defined dot-blot spots (Figure 2) while minimizing the amount of protein required per antigen position, to a mere 400 ng. We adopted a simple double-spotting technique at each antigen position using a micropipette to achieve our desired results. It is important to note that this double-step technique was necessary only after we reconstituted the lyophilized proteins to a concentration of 250 ng/µL, as per the manufacturer’s instructions. In order to produce well-defined spots of approximately 0.4 mm in diameter as shown in Figure 2, it was necessary to employ a double spotting technique using a micropipette. Specifically, 0.8 µL of the protein solution was loaded onto the membrane twice. Attempts to spot 400 ng of protein using a single 1.6 µL load resulted in spots with a larger diameter than the ideal 0.4 mm, and more importantly, the Ponceau staining of these larger spots appeared diffuse (not shown). These diffused spots were not suitable for our design as they would likely decrease the overall sensitivity of the assay by dispersing the final chromogenic signal over a larger area. However, it is possible to reconstitute lyophilized proteins at a higher concentration (e.g., 500 ng/µL), which would allow for the spotting of the same amount of protein (400 ng) within a 0.4 mm diameter spot using a single 0.8 µL load. This alternative would have the added advantage of significantly reducing the preparation time required for the strips.

Initially, we attempted to detect immunoreactivity on dot-blot strips using a plasma pool from COVID-19-positive individuals in combination with both anti-IgG and anti-IgM secondary antibodies, each on a separate dot-blot strip. However, this approach revealed that the use of anti-IgM did not contribute any additional value to the assay, as no spots corresponding to the RBD or nucleocapsid proteins antigens turned purple (not shown). Moreover, our results from using anti-IgM antibodies for immunoblot detection revealed only a faint nucleocapsid protein band and the absence of any RBD protein band (Figure 1). These results were anticipated as the COVID-19-positive samples included in the plasma pool were collected several days or a few weeks after the end of the infection, and therefore, most of them fall outside the window of IgM detection. As a result, we decided to rely solely on anti-IgG detection for the validation of the ELISA dot-blot assay. This decision not only simplified the final assay but also further reduced its overall cost.

It is worth mentioning that the recombinant proteins used in the development of this assay were produced in mammalian cells (i.e., HEK-293). We are aware that recombinant SARS-CoV-2 proteins expressed in prokaryotic cells, such as *E. coli*, are more common and tend to have a lower cost in the global market, in contrast to those expressed using eukaryotic cells platforms such as mammalian, insect, or yeast cells. Nonetheless, we made a deliberate choice to use proteins expressed in eukaryotic cells as at the time of our experimental design, there was an absence of information on any structural variations that could arise from expression in bacteria, since post-translational modifications of both the RBD and nucleocapsid proteins in beta-coronaviruses play crucial roles in determining the structure and immunoreactivity of these proteins. For example, previous studies have shown that arginine/serine phosphorylation positions within the nucleocapsid protein are structurally functional in closely related SARS-CoV [11–13]. Furthermore, Wu et al. [12] developed a murine phosphospecific antibody against an epitope within the serine-rich region of SARS-CoV’s nucleocapsid protein. The RBD protein, on the other hand, contains several glycosylation sites that directly impact the production of neutralizing antibodies against SARS-CoV-2 [14,15]. Therefore, both of these antigens contain post-transcriptional modification sites that play a role in immunoreactivity, and thus, their modification status (i.e., glycosylated vs non-glycosylated or phosphorylated vs non-phosphorylated) could potentially change the sensitivity and specificity of immunoassays developed to detect seroconversion. While at the beginning of this project we had little to no direct evidence on whether these post-translational modifications could had an effect on the sensitivity and specificity of the assay, out of an abundance of caution, we made the decision to use the expression platform that would produce the antigens that are structurally closer to the viral protein produced by infected human cells. Therefore, we chose recombinant antigens expressed in a eukaryotic platform as the most suitable option for the immunoassay, despite their higher costs. However, with the rapid advancement of technology in the field of COVID-19, researchers have recently developed at least one recombinant RBD protein expressed in *E. coli* that is functional and immunoreactive to COVID-19 convalescent plasma [16]. This kind of development could lead to more reasonably priced recombinant antigens on the market in the near future, further reducing the cost of this type of immunoassay.

It is also imperative to note that only a blotting-specific TMB (3,3′,5,5′-tetramethylbenzidine) can be employed with this type of membrane-based assay. A blotting-specific TMB creates an insoluble chromogenic precipitate that adheres to the membrane, while traditional TMB solutions used in “wet” ELISAs generate a soluble chromogen that is washed away, resulting in little to no chromogenic signal left on the membrane. Other blotting-specific chromogenic substrates may also be used; however, when we attempted to develop our dot-blot strips using chloronaphthol, we had some success (not shown) but the chromogenic signal observed was much weaker. Therefore, we cannot recommend utilizing chloronaphthol in this assay. Lastly, we also tested our dot-blot strips using chemiluminescence detection, which yielded very clear results (not shown); however, this approach goes against the principle of our effort as it imposes the use of an expensive detection instrument or additional steps and materials incorporated into the workflow.

It is worth noting that of the anti-SARS-CoV-2 immunoreactive samples detected by our ELISA dot-blot test and confirmed by either CLIA or ELISA, 19% of them were found to be immunoreactive against the nucleocapsid protein alone, while the remaining 81% displayed immunoreactivity against both the RBD and nucleocapsid proteins. Notably, none of the positive samples tested produced an RBD protein signal alone. Additionally, when using the COVID-19-positive plasma pool for immunoblotting, a nucleocapsid protein band was observed, but no RBD protein signal was detected (Figure 1). These results appear to be in contrast to data from other studies that have used COVID-19 convalescent plasma, which indicate that anti-nucleocapsid protein immunoreactivity diminishes much faster than anti-RBD protein immunoreactivity [17]. A possible explanation for a stronger or longer-lasting anti-nucleocapsid protein immunoreactivity among the samples used is the molecular size of the selected recombinant antigens. A heavier (47 kDa) nucleocapsid protein provides a much larger substrate for potential epitopes than the lighter (27 kDa) RBD protein.

In any case, the results of differential immunoreactivity against RBD versus nucleocapsid protein in human plasma suggest that the test we developed could be performed using nucleocapsid protein as a single antigen while maintaining the same sensitivity and specificity. However, we recommend that this assay is always performed with the two selected antigens, since the positive selection criteria we developed use the chromogenic signal from both antigens (see Materials and Methods). Furthermore, the use of immunoreactivity against nucleocapsid and RBD proteins together could provide additional epidemiological surveillance information in some places where vaccination against SARS-CoV-2 is already widespread. In this respect, it should also be noted that all COVID-19-positive samples were collected months before vaccines were available. Therefore, both anti-nucleocapsid and anti-RBD protein immunoreactivity can only be attributed to the COVID-19 infection itself. Currently, anti-RBD immunoreactivity is also found among individuals inoculated with vaccines that use the full-length Spike protein as a single antigen. These include the most commonly used vaccines such as viral vector- and mRNA-based vaccines. No vaccines using nucleocapsid protein as an antigen have been approved by any major drug or healthcare regulatory agency to date. Thus, inoculation with the most used vaccines would lead to anti-RBD protein immunoreactivity alone, while immunoreactivity against nucleocapsid protein in vaccinated individuals could only be explained due to a previous COVID-19 infection. However, the anti-nucleocapsid protein immunoreactivity would not work as a post-infection marker in places where inactivated or attenuated virus vaccines are introduced, as these drugs contain all viral antigens.

Unfortunately, we did not have access to pre-pandemic samples confirmed to be seropositive for traditionally endemic coronaviruses such as HCoV-HKU1 and HCoV-OC43. Furthermore, no data on seroprevalence for these viruses has been published for the Costa Rican population. Nonetheless, these traditionally endemic coronaviruses are the most common cause of diarrhea among Costa Rican children, accounting for up to 38% of all cases [18,19]. As a result, it is likely that the seroprevalence for these traditionally endemic coronaviruses is high among Costa Rican adults, and seropositive individuals must be present among the 20 pre-pandemic samples used. These limitations also influenced our antigen selection. In the case of RBD, the high divergence of its structure across the human coronaviruses makes it an ideal antigen for specificity with little to no potential for cross-reactivity among these related viruses [20,21]; nonetheless, our results show that used as a single antigen, the RBD protein would have made our assay’s sensitivity to be too low for any practical purposes. Therefore, it is necessary to combine it with another antigen such as the chosen nucleocapsid protein, even though it has its own limitations. For example, the literature has reported some level of unspecific cross-reactivity against the full SARS-CoV-2’s nucleocapsid protein in pre-pandemic human plasma samples [22–24]. This type of cross-reactivity against the nucleocapsid proteins of different coronaviruses is expected because of a few conserved sequences among these homologous proteins. Of particular importance, the nucleocapsid proteins of beta-coronaviruses contain a 12-residue sequence (PRWYFYYLGTGP) that is perfectly conserved among many examples of the clade, including SARS-CoV, SARS-CoV-2, HCoV-HKU1, and HCoV-OC43. A shorter form of the same protein sequence (FYYLGTGP) is also shared among those beta-coronaviruses and the endemic alpha-coronaviruses HCoV-NL63 and HCoV-229E. Furthermore, this shared sequence has been identified as a part of an epitope involved in T-cell- and B-cell-based immunity against SARS-CoV-2 [24–26]. This cross-reactivity is a concern for all forms of immunoassays that use the nucleocapsid protein to detect antibodies against coronaviruses, including our ELISA dot-blot test. Furthermore, given the position of this conserved sequence towards the middle of the nucleocapsid proteins, a partial antigen with an amino end deletion that removes the conserved epitope would represent a significantly smaller antigen and could potentially affect the assay’s sensitivity. In any case, to our knowledge, there are no commercially available truncated SARS-CoV-2’s nucleocapsid proteins lacking this conserved epitope. Regardless of the theoretical implications of this conserved epitope, quantification of antibodies that react against the nucleocapsid protein of SARS-CoV-2 shows that their titers are drastically lower among pre-pandemic samples than in the plasma of COVID-19 convalescent individuals. In some cases, titer levels in pre-pandemic samples and COVID-19 convalescent plasma differ by an order of magnitude [22–24]. For example, using a SARS-CoV-2’s nucleocapsid protein IgG ELISA, Pajenda et al. [25] did not detect immunity against this antigen among 240 healthcare workers until a COVID-19 outbreak infected at least 24 of them and caused their seroconversion. Therefore, the titers of unspecific cross-reactive antibodies against the nucleocapsid protein of other coronaviruses seem to fall below the lower detection limit of some immunoassays, including our dot-blot ELISA. This point is demonstrated by the absence of any unspecific chromogenic signal among our 20 pre-pandemic plasma samples. Additionally, it is worth mentioning that the 20 pre-pandemic human plasma samples were obtained from individuals who were previously diagnosed with dengue, zika, or chikungunya; thus, our ELISA dot-blot test does not show any cross-reactivity with antibodies against these arboviruses.

The implementation of a manufacturing center for the dot-blot strips would help to simplify the logistics of this test. These manufacturing centers would allow the shipment of ready-to-use strips to the communities where the tests must be performed. However, to achieve this goal, the dot-blot strips must be stable enough to be transported under commonly used conditions such as cold or freezing temperatures. Our initial tests using dried dot-blot strips demonstrated that they lose the ability to detect immunoreactivity within 24 h of storage even under freezing conditions (not shown). These results mean that virgin dot-blot strips should never be kept completely dry for more than a couple of hours. For that reason, we also tested the stability of the dot-blot strips when stored in sealed plastic sleeves with enough PBS-Tween to keep them wet. For this test, we stored the dot-blot strips for 3 or 7 days at 4 or −10°C, two commonly used storage conditions employed by most distribution cold chains. Both storage conditions (4 and −10°C) fully preserve the functionality of the strips after 3 days (Figure 3). When the storage period was extended to 7 days, the −10°C condition fully preserved the functionality of the strip; however, the strip stored at 4°C lost its ability to detect anti-RBD immunoreactivity (Figure 3). These results imply that the dot-blot strips can be shipped from a manufacturing facility using very basic cold-chain conditions such as regular refrigeration or coolers with icepacks if the shipment takes 3 days or less. For storage or shipment periods of up to 7 days, freezing conditions are required.

While the assay developed requires some basic laboratory equipment, including micropipettes, a centrifuge, and a shaker; the cost and availability of this equipment is much more accessible than the instruments necessary for commercial ELISA or CLIA platforms or even a simple colorimetric plate reader. Our test also provides a reasonable turnaround time of approximately one hour. Preparation of the dot-blot strips takes about one extra hour. Compared to LFIA tests, this ELISA dot-blot requires more time, human labor, as well as the equipment already mentioned; however, the total cost of the materials for this ELISA dot-blot is a fraction of any LFIA test available in the global market. According to our calculations, the cost of the recombinant proteins for this assay amounts to approximately $3.6 per test and the total material cost falls under $4.5. Meanwhile, the price of a COVID-19 IgG LFIA test currently starts at around $20. We calculated that the final cost of running our assay would be less than half the investment of using a commercial LFIA. However, the economic benefits can only be accurately defined once associated costs such as shipping, taxes, duties, labor, and infrastructure are considered for each country and setting. For that reason, we recommend that any epidemiological surveillance effort that plans to use our assay begins with a financial and technical evaluation of all associated costs and requirements to select the most affordable or otherwise ideal option.

In short, we developed an immunoassay to detect anti-SARS-CoV-2 antibodies using very basic laboratory equipment, at a price that is much lower than commercial LFIAs. This assay may be an affordable and simple option for COVID-19 seroprevalence surveillance, especially in places where clinical laboratories are not available and resources for this type of evaluation are limited.

## Figures and Tables

**Figure A1:**
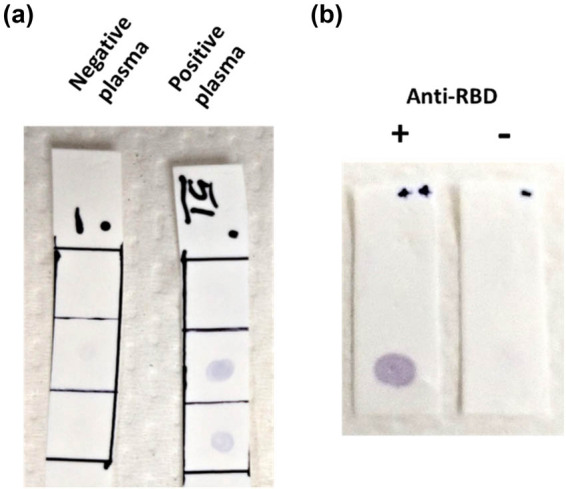
Full-length dot-blot strip images of the results shown in Figure 2: (a) full-length picture of the dot-blots strips shown in Figure 2a, and (b) full-length picture of the dot-blots strips shown in Figure 2b.

**Figure A2:**
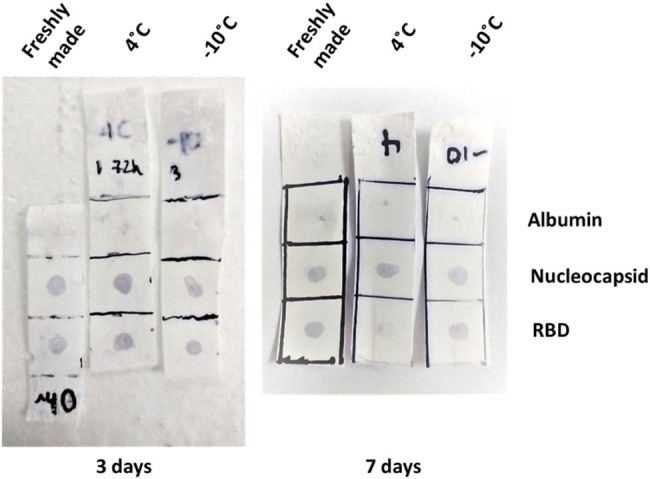
Full-length dot-blot strip images of the results shown in Figure 3 for dot-blot strips stored for 3 or 7 days at 4 or −10°C.
